# A study to assess the correlation between plasma, oral fluid and urine concentrations of flunixin meglumine with the tissue residue depletion profile in finishing-age swine

**DOI:** 10.1186/s12917-020-02429-w

**Published:** 2020-06-22

**Authors:** Jessica L. Bates, Locke A. Karriker, Suzanne M. Rajewski, Zhoumeng Lin, Ronette Gehring, Mengjie Li, Jim E. Riviere, Johann F. Coetzee

**Affiliations:** 1 Swine Medicine Education Center, Veterinary Diagnostic and Production Animal Medicine, College of Veterinary Medicine, Iowa State University, Ames, Iowa 50011 USA; 2 Analytical Chemistry Services, Veterinary Diagnostic and Production Animal Medicine, College of Veterinary Medicine, Iowa State University, Ames, Iowa 50011 USA; 3 Institute of Computational Comparative Medicine (ICCM), Department of Anatomy and Physiology, College of Veterinary Medicine, Kansas State University, 1800 Denison Avenue, P200 Mosier Hall, Manhattan, KS 66506 USA; 4 Present Address: Ronette Gehring, Institute for Risk Assessment Sciences, Division of Toxicology and Pharmacology, Utrecht University, Utrecht, The Netherlands; 5 Present Address: Department of Pharmaceutical Sciences, School of Pharmacy and Pharmaceutical Sciences, University at Buffalo, Buffalo, NY 14260 USA

**Keywords:** Flunixin meglumine, Food safety, Pharmacokinetics, Physiologically based pharmacokinetic (PBPK) model, Swine, Tissue residue

## Abstract

**Background:**

Flunixin meglumine (FM) was investigated for the effectiveness of plasma, oral fluid, and urine concentrations to predict tissue residue depletion profiles in finishing-age swine, along with the potential for untreated pigs to acquire tissue residues following commingled housing with FM-treated pigs. Twenty pigs were housed in groups of three treated and one untreated control. Treated pigs received one 2.2 mg/kg dose of FM intramuscularly. Before treatment and at 1, 3, 6, 12, 24, 36, and 48 h (h) after treatment, plasma samples were taken. At 1, 4, 8, 12 and 16 days (d) post-treatment, necropsy and collection of plasma, urine, oral fluid, muscle, liver, kidney, and injection site samples took place. Analysis of flunixin concentrations using liquid chromatography/tandem mass spectrometry was done. A published physiologically based pharmacokinetic (PBPK) model for flunixin in cattle was extrapolated to swine to simulate the measured data.

**Results:**

Plasma concentrations of flunixin were the highest at 1 h post-treatment, ranging from 1534 to 7040 ng/mL, and were less than limit of quantification (LOQ) of 5 ng/mL in all samples on Day 4. Flunixin was detected in the liver and kidney only on Day 1, but was not found 4–16 d post-treatment. Flunixin was either not seen or found less than LOQ in the muscle, with the exception of one sample on Day 16 at a level close to LOQ. Flunixin was found in the urine of untreated pigs after commingled housing with FM-treated pigs. The PBPK model adequately correlated plasma, oral fluid and urine concentrations of flunixin with residue depletion profiles in liver, kidney, and muscle of finishing-age pigs, especially within 24 h after dosing.

**Conclusions:**

Results indicate untreated pigs can be exposed to flunixin by shared housing with FM-treated pigs due to environmental contamination. Plasma and urine samples may serve as less invasive and more easily accessible biological matrices to predict tissue residue statuses of flunixin in pigs at earlier time points (≤24 h) by using a PBPK model.

## Background

Flunixin meglumine (FM) (Banamine-S®, Merck Animal Health, Madison, NJ) is approved as an intramuscular (IM) injection for pyrexia control associated with swine respiratory disease [1] and is available for intravenous (IV) and oral administration in other livestock species [2]. It is labeled for use in the United States for beef cattle, dairy cattle, horses and swine [2, 3]. It has been utilized with pharmacokinetic parameters assessed in numerous species [4–16]. Pharmacokinetic properties of FM have been described in smaller non-breeding aged swine with weights ranging from 20 kg to 40 kg [3, 17, 18], mature swine (152–168 kg) [19], and sows (187–259 kg) [20], but scant research findings exist on finishing-age swine (90–130 kg).

Finishing-age swine tissues as they relate to the proximity to human consumption set them apart as important with regard to treatment decisions. A safe, healthy product for pork consumers is paramount. FM is regularly utilized as an adjunctive treatment in respiratory disease of finishing swine. Respiratory diseases rank among the most serious disease problems and pose remarkable problems to swine production today [21]. However, these threats also open opportunities for interventions through antimicrobials and adjunctive treatments such as FM.

Details of flunixin drug tissue residues and alternative methods of residue detection in finishing-age swine is limited. The labeled tissue withdrawal period for FM in swine is 12 d [2]. Traditional plasma and carcass sampling provides drug residue measurement in conjunction with emerging means, such as analysis of oral fluids and urine. Oral fluids are already utilized efficiently to detect diseases [22] and antimicrobial residues [23] in swine. Coordinating sampling methodology should help develop a less-invasive, more easily administered antemortem monitoring procedure.

Searches for peer-reviewed literature show limited research on carcass residue effects of FM in finishing-age swine and their association with other sampling methods. In the only swine study found, Magyar and Glavits [24] demonstrated FM had significantly (*p* < 0.05) worse carcass lesions than meloxicam, grossly visible out to 15 d after administration. In other species, flunixin is the second-most common residue violation in cull dairy cattle [25]. It is labeled for IV injection and requires a withdrawal period of 4 d in cattle (excluding veal calves). Kissell et al. [11] demonstrated the extended duration of tissue and milk residues when FM was given by IM and SQ in cattle. In addition, environmental contamination with drug residues represents another serious consideration. In a study by Popot et al. [26], horses given FM while staying in the same stall with no daily bedding change had flunixin levels in urine that were detectably extended. It was concluded this was likely from ingestion of bedding contaminated by urine because renal clearance is the main route of flunixin excretion. These factors need to be kept in mind in pork residues as well.

The objectives of this study were to: 1) determine the potential correlation of plasma, oral fluid, and urine concentrations of flunixin with its residue depletion kinetic profiles in edible tissues of finishing-age pigs, 2) evaluate the potential for flunixin exposure of untreated pigs by treated pigs due to environmental contamination, and 3) evaluate the effectiveness of describing plasma, urine and oral fluid flunixin levels as potential biomarkers of tissue residue statuses in swine. The objectives of this study aim to advance understanding of flunixin tissue residues in swine and provide novel testing methods to limit carcass tissue residues.

## Results

### Plasma pharmacokinetics

The concentrations of flunixin and its metabolite 5-hydroxyflunixin in the plasma of finishing-age swine after IM administration of FM at 2.2 mg/kg are presented in Tables 1 and 2, respectively. Plasma concentration data are reported as a number or <LOQ. Over 90% of the <LOQ for the plasma samples were not detected, but there were seven samples above the LOD of 3 ng/mL and below the LOQ of 5 ng/mL; and four of these seven samples belonged to the control group where all of the other time points were not detected. The other three samples were from animals 269, 258, and 272, and might due to possible contamination at some point in the process. These seven samples prompted us to present <LOQ or a number in Tables 1 and 2.

**Table 1:** Plasma flunixin concentrations (ng/mL) after intramuscular administration of flunixin meglumine at 2.2 mg/kg in finishing-age swine

Group	G1 (Day 1)				G2 (Day 4)				G3 (Day 8)				G4 (Day 12)				G5 (Day 16)			
**Pig / h**	**260**	**261**	**270 (C)**	**273**	**262**	**264**	**269**	**274 (C)**	**258**	**266 (C)**	**268**	**272**	**263**	**267**	**271**	**275 (C)**	**257**	**259**	**265 (C)**	**276**
**0 h**	<LOQ	<LOQ	<LOQ	<LOQ	<LOQ	<LOQ	<LOQ	<LOQ	<LOQ	<LOQ	<LOQ	<LOQ	<LOQ	<LOQ	<LOQ	<LOQ	<LOQ	<LOQ	<LOQ	<LOQ
**1 h**	1534	4797	<LOQ	7040	4645	3365	2677	<LOQ	4093	<LOQ	1927	3791	2973	1857	5781	<LOQ	3293	2593	<LOQ	2969
**3 h**	863	2337	<LOQ	2955	1925	2049	1524	<LOQ	2278	<LOQ	1314	3262	3926	844	3149	<LOQ	1512	1423	<LOQ	2121
**6 h**	667	1015	<LOQ	1839	739	901	625	<LOQ	1217	<LOQ	633	1145	1352	439	1651	<LOQ	753	931	<LOQ	2262
**12 h**	495	442	<LOQ	321	325	352	238	<LOQ	492	<LOQ	314	563	403	187	490	<LOQ	284	327	<LOQ	664
**24 h**	113	51	<LOQ	53	63	88	31	<LOQ	55	<LOQ	73	59	33	49	83	<LOQ	71	49	<LOQ	163
**36 h**					21	21	7	<LOQ	15	<LOQ	54	19	10	26	19	<LOQ	38	18	<LOQ	63
**Day 2**					21	8	<LOQ	<LOQ	<LOQ	<LOQ	25	<LOQ	<LOQ	11	6	<LOQ	9	<LOQ	<LOQ	<LOQ
**Day 4**					<LOQ	<LOQ	<LOQ	<LOQ												
**Day 8**									<LOQ	<LOQ	<LOQ	<LOQ								
**Day 12**													<LOQ	<LOQ	<LOQ	<LOQ				
**Day 16**																	<LOQ	<LOQ	<LOQ	<LOQ

Note: Concentrations that were below the level of quantification (LOQ) (5 ng/mL) were designated “LOQ”. Untreated, control pigs used to assess the potential for environmental contamination were designated (C)

**Table 2:** Plasma 5-hydroxyflunixin concentrations (ng/mL) after intramuscular administration of flunixin meglumine at 2.2 mg/kg in finishing-age swine

Group	G1 (Day 1)				G2 (Day 4)				G3 (Day 8)				G4 (Day 12)				G5 (Day 16)			
**Pig / h**	**260**	**261**	**270 (C)**	**273**	**262**	**264**	**269**	**274 (C)**	**258**	**266 (C)**	**268**	**272**	**263**	**267**	**271**	**275 (C)**	**257**	**259**	**265 (C)**	**276**
**0 h**	<LOQ	<LOQ	<LOQ	<LOQ	<LOQ	<LOQ	<LOQ	<LOQ	<LOQ	<LOQ	<LOQ	<LOQ	<LOQ	<LOQ	<LOQ	<LOQ	<LOQ	<LOQ	<LOQ	<LOQ
**1 h**	61	<LOQ	<LOQ	101	<LOQ	207	124	<LOQ	131	<LOQ	127	135	<LOQ	45	127	<LOQ	105	108	<LOQ	321
**3 h**	<LOQ	<LOQ	<LOQ	102	11	132	96	<LOQ	163	<LOQ	113	174	570	46	105	<LOQ	84	82	<LOQ	284
**6 h**	<LOQ	<LOQ	<LOQ	83	<LOQ	<LOQ	52	<LOQ	100	<LOQ	55	86	<LOQ	27	89	<LOQ	46	19	<LOQ	205
**12 h**	7	<LOQ	<LOQ	51	<LOQ	17	24	<LOQ	53	<LOQ	31	64	<LOQ	21	29	<LOQ	22	35	<LOQ	69
**24 h**	<LOQ	<LOQ	<LOQ	<LOQ	<LOQ	<LOQ	<LOQ	<LOQ	<LOQ	<LOQ	5	<LOQ	<LOQ	<LOQ	<LOQ	<LOQ	6	6	<LOQ	12
**36 h**					<LOQ	<LOQ	<LOQ	<LOQ	<LOQ	<LOQ	<LOQ	<LOQ	<LOQ	<LOQ	<LOQ	<LOQ	<LOQ	<LOQ	<LOQ	8
**Day 2**					<LOQ	<LOQ	<LOQ	<LOQ	<LOQ	<LOQ	<LOQ	<LOQ	<LOQ	<LOQ	<LOQ	<LOQ	<LOQ	<LOQ	<LOQ	<LOQ
**Day 4**					<LOQ	<LOQ	<LOQ	<LOQ												
**Day 8**									<LOQ	<LOQ	<LOQ	<LOQ								
**Day 12**													<LOQ	<LOQ	<LOQ	<LOQ				
**Day 16**																	<LOQ	<LOQ	<LOQ	<LOQ

Note: Concentrations that were below the level of quantification (LOQ) (5 ng/mL) were designated “LOQ”. Untreated, control pigs used to assess the potential for environmental contamination were designated (C)

At pre-treatment of 0 h, the concentrations of both flunixin and 5-hydroxyflunixin in all plasma samples were less than LOQ of 5 ng/mL. Flunixin concentrations were the highest at 1 h post-treatment, with a range of 1534–7040 ng/mL, indicating a high variability in the plasma concentration of flunixin after IM administration. From 1 h to 48 h post-treatment, the concentration of flunixin continued to drop. At 96 h post-treatment, the concentration of flunixin was less than LOQ in all collected plasma samples.

Compared to flunixin, the concentrations of 5-hydroxyflunixin in the plasma had relatively higher variability (Table 2). At 1 h after treatment, three of the fifteen plasma samples from FM-treated swine had 5-hydroxyflunixin concentrations less than LOQ of 5 ng/mL; and the levels ranged from 61 to 321 ng/mL in the remaining twelve samples. Concentrations of 5-hydroxyflunixin in the plasma fell over time. At 48 h post-treatment, the concentration of 5-hydroxyflunixin was less than LOQ in all collected plasma samples.

### Tissue residue depletion

Concentrations of flunixin and 5-hydroxyflunixin in the tissues of finishing-age swine after IM administration of FM at 2.2 mg/kg are presented in Tables 3 and 4, respectively. On Day 1 (at 24 h) after treatment, concentrations of flunixin fell in the range of 77.5 to 160.1 ng/g in kidney, from 98.6 to 326.8 ng/g in liver (higher than the tolerance of 30 ng/g in liver), and were either <LOQ or not found in the muscle. From Day 2 to Day 16, all liver, kidney, and muscle samples showed flunixin concentrations less than LOQ, except one muscle sample on Day 16 that was 9.8 ng/g, which is lower than the tolerance of 25 ng/g in muscle of swine. In the injection site on Day 1, flunixin concentrations ranged widely, from 7.5 to 40,143.4 ng/g. Flunixin was still found in one of the three treated injection site samples on Day 12 after treatment, but flunixin was less than LOQ in all samples on Day 16 after dosage. Similar to flunixin, 5-hydroxyflunixin was found on Day 1, but not on Days 2–16 in kidney and liver. In the muscle, 5-hydroxyflunixin did not appear at any sampling period. In the injection site, 5-hydroxyflunixin was either not found or less than LOQ from Day 1 to Day 16 after treatment.

**Table 3:** Tissue flunixin concentrations (ng/g) in liver, kidney, semitendinosus/semimembranosus muscle, and injection site after intramuscular administration of flunixin meglumine at 2.2 mg/kg in finishing-age swine

Group	Pig ID	Liver	Kidney	Muscle	Injection Site
G1 (Day 1)	260	326.8	160.1	NF	40,143.4
	261	105.8	77.5	<LOQ	7.5
	270 (C)	NF	NF	NF	NF
	273	98.6	115.2	<LOQ	887.7
G2 (Day 4)	262	NF	NF	NF	13.4
	264	NF	NF	NF	NF
	269	NF	NF	NF	<LOQ
	274 (C)	NF	NF	NF	NF
G3 (Day 8)	258	NF	NF	NF	NF
	266 (C)	NF	NF	NF	NF
	268	NF	NF	NF	<LOQ
	272	NF	NF	NF	NF
G4 (Day 12)	263	NF	NF	NF	NF
	267	NF	NF	NF	28.9
	271	NF	NF	NF	NF
	275 (C)	NF	NF	NF	NF
G5 (Day 16)	257	NF	NF	NF	<LOQ
	259	NF	NF	9.8	NF
	265 (C)	NF	NF	NF	<LOQ
	276	NF	NF	NF	NF

Note: Concentrations that were below the level of quantification (LOQ) were designated “LOQ”. For this assay the LOQ was 5 ng/g. If no flunixin levels were detected, the samples were designated “NF”. Untreated, control pigs used to assess the potential for environmental contamination were designated (C)

**Table 4:** Tissue 5-hydroxyflunixin concentrations (ng/g) in liver, kidney, semitendinosus/semimembranosus muscle, and injection site after intramuscular administration of flunixin meglumine at 2.2 mg/kg in finishing-age swine

Group	Pig ID	Liver	Kidney	Muscle	Injection Site
G1 (Day 1)	260	303.5	76.2	NF	<LOQ
	261	94.1	106.1	NF	NF
	270 (C)	NF	NF	NF	NF
	273	87.8	47.2	NF	<LOQ
G2 (Day 4)	262	NF	NF	NF	NF
	264	NF	NF	NF	NF
	269	NF	NF	NF	NF
	274 (C)	NF	NF	NF	NF
G3 (Day 8)	258	NF	NF	NF	<LOQ
	266 (C)	NF	NF	NF	NF
	268	NF	NF	NF	NF
	272	NF	NF	NF	NF
G4 (Day 12)	263	NF	NF	NF	NF
	267	NF	NF	NF	NF
	271	NF	NF	NF	NF
	275 (C)	NF	NF	NF	NF
G5 (Day 16)	257	NF	NF	NF	<LOQ
	259	NF	NF	NF	NF
	265 (C)	NF	NF	NF	NF
	276	NF	NF	NF	NF

Note: Concentrations that were below the level of quantification (LOQ) were designated “LOQ”. For this assay the LOQ was 5 ng/g. If no 5-hydroxyflunixin levels were detected, the sample was designated “NF”. Untreated, control pigs used to assess the potential for environmental contamination were designated (C)

### Concentrations in urine and Oral fluids

Flunixin and 5-hydroxyflunixin concentrations in urine and oral fluid samples from finishing-age swine after IM administration of FM at 2.2 mg/kg are reported in Table 5. Flunixin showed in the urine up to Day 4 after treatment, but it was either not found or less than LOQ after 8 d and 16 d. On Day 1, 5-hydroxyflunixin was found in the urine, but it was either less than LOQ or not found from Days 2–16. Concentrations of flunixin and 5-hydroxyflunixin in the pen-level oral fluid samples varied greatly. Flunixin was quantifiable on Days 1, 4, and 12, but not on Days 8 and 16. Meanwhile, 5-hydroxyflunixin was quantifiable on Days 1, 4, 12, and 16 at low levels (10.6–21.0 ng/mL), but not on Day 8.

**Table 5:** Flunixin and 5-hydroxyflunixin concentrations (ng/mL) in urine and oral fluids after intramuscular administration of flunixin meglumine at 2.2 mg/kg in finishing-age swine

Group	Pig ID	Urine		Pen-Level Oral Fluids	
		Flunixin	5-hydroxyflunixin	Flunixin	5-hydroxyflunixin
G1 (Day 1)	260	9459.1	1295.0	168.6	21.0
	261	3762.5	972.0		
	270 (C)	231.4	16.5		
	273	3032.0	724.4		
G2 (Day 4)	262	60.3	<LOQ	16.8	10.9
	264	22.7	<LOQ		
	269	35.5	<LOQ		
	274 (C)	11.6	<LOQ		
G3 (Day 8)	258	<LOQ	<LOQ	<LOQ	<LOQ
	266 (C)	<LOQ	NF		
	268	<LOQ	NF		
	272	<LOQ	NF		
G4 (Day 12)	263	NF	NF	12.3	11.3
	267	<LOQ	NF		
	271	<LOQ	NF		
	275 (C)	<LOQ	NF		
G5 (Day 16)	257	NF	NF	<LOQ	10.6
	259	NF	NF		
	265 (C)	NF	NF		
	276	NF	NF		

Note: Oral fluids were a group sample from all four pigs in the group at the day of necropsy. Concentrations that were below the level of quantification (LOQ) were designated “LOQ”. For this assay the LOQ was 5 ng/mL. If no flunixin or 5-hydroxyflunixin levels were detected, the sample was designated “NF”. Untreated, control pigs used to assess the potential for environmental contamination were designated (C)

### PBPK model simulation

**Fig. 1:**
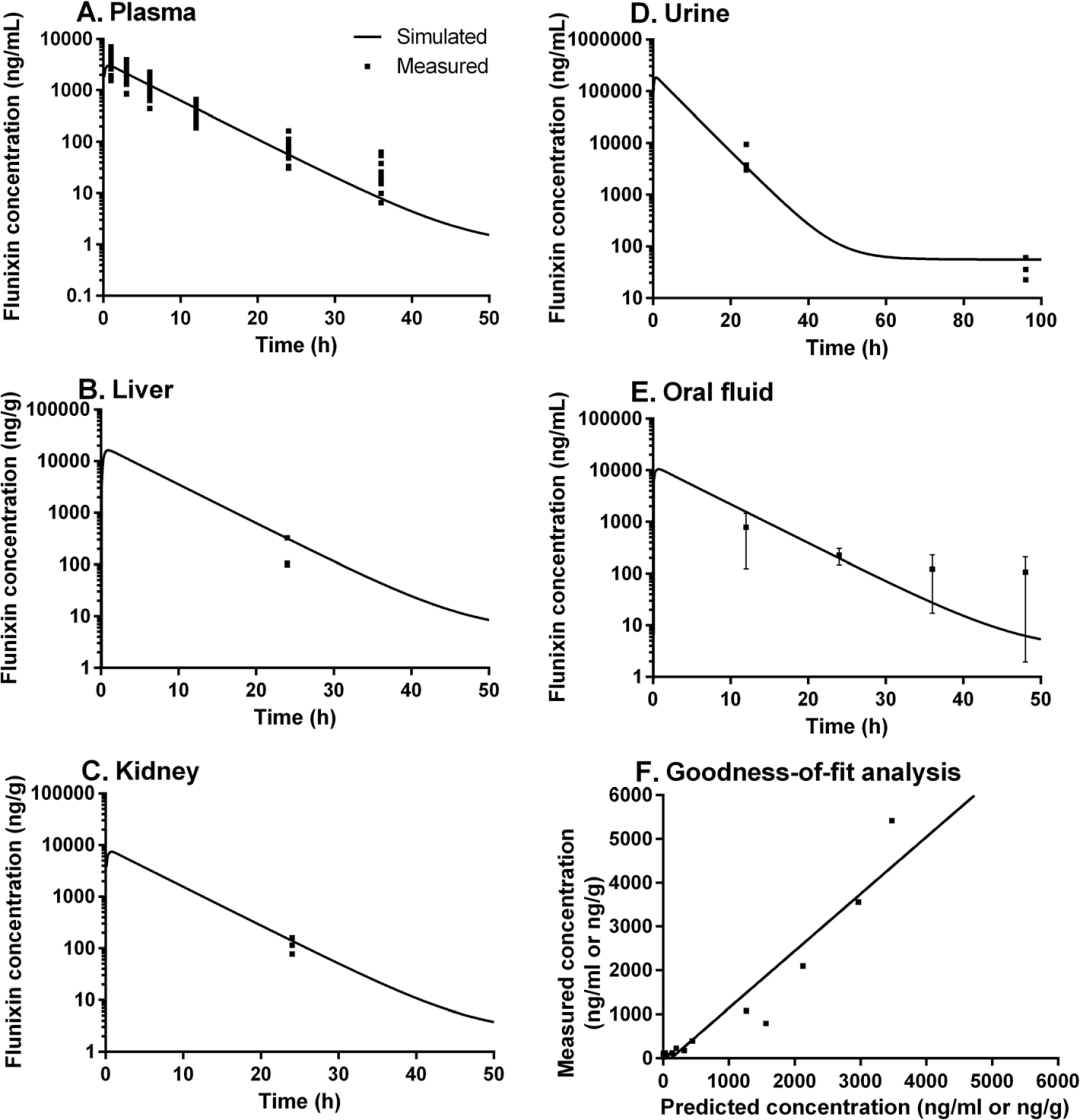
Model calibration results. Comparisons of model predictions (solid lines) and observed data (squares) for flunixin concentrations in the plasma (**a**), liver (**b**), kidneys (**c**), urine (**d**), and oral fluids (**e**) of finishing-age pigs following intramuscular injection with 2.2 mg/kg flunixin meglumine. Only observed data that were higher than limit of quantification (LOQ = 5 ng/ml) are shown. Measured flunixin concentrations in the muscle at 24 h were < LOQ in two animals and not found in one animal, which was consistent with the simulated data (4.70 ng/g) that was <LOQ. At 96 h after injection, observed flunixin concentrations in liver, kidneys, or muscle were all not found, which also corresponded to simulated results (5.03, 2.26, 0.08 ng/g in liver, kidneys, and muscle, respectively). Panel **f** represents the regression analysis result between measured and simulated data. The regression coefficient was *R*^*2*^ = 0.91, suggesting high overall goodness-of-fit

PBPK model simulations of plasma, tissue, oral fluid, and urine concentrations of flunixin were compared with measured data in pigs exposed to 2.2 mg/kg FM via IM administration (Fig. 1). The model successfully simulated the observed flunixin concentrations in the plasma, especially within 24 h after treatment (Fig. 1a). At later time points (≥48 h), observed data are not shown because the majority of the observed values in plasma were lower than LOQ (Tables 1 and 2). To be more specific, at 48 h, observed flunixin levels were < LOQ in six out of the twelve experimental animals, and at ≥96 h observed values were < LOQ in all animals (Table 1). These experimentally-measured results were also consistent with the PBPK model-simulated values, which were < LOQ (e.g., 1.85 ng/ml at 48 h) at all these later time points.

After calibration with observed plasma concentration data, the PBPK model was directly employed to simulate liver flunixin concentrations and it adequately simulated the concentration at 24 h (Fig. 1b). At ≥96 h, flunixin was not found in the liver. These numbers were consistent with model-predicted results that were either close to or < LOQ in the liver at these studied time points (≥96 h). In the original PBPK model in cattle [27], the kidney/plasma partition coefficient for flunixin was not optimized due to insufficient data. In the present study, we estimated this parameter by fitting to the observed kidney concentration data at 24 h (Fig. 1c). By using this estimated partition coefficient, the model-simulated concentrations of flunixin in kidney at ≥96 h were all much lower than LOQ, which corresponded to the experimentally-observed data that were all not found. In addition, model-simulated flunixin levels in muscle at all time points were below LOQ, which correlated with the observed data that were either <LOQ or not found except on Day 16 in Animal 259, where the muscle concentration was 9.8 ng/g (Table 3). It is worth mentioning that concentrations of flunixin found in all the tissues beyond Day 12 (the labeled tissue withdrawal period for FM in pigs) were less than the established tolerance levels of flunixin in swine, which are 30 and 25 ng/g in liver and muscle, respectively [28].

## Discussion

The present study reports original experimental data on the concentrations of flunixin and its metabolite 5-hydroxyflunixin in plasma, liver, kidney, muscle, injection site, urine, and oral fluid of finishing-age swine after IM administration of FM at the label dose of 2.2 mg/kg. The results fill in the data gaps on the plasma pharmacokinetics and tissue residue depletion profile of flunixin and 5-hydroxyflunixin in finishing-age swine after IM administration. Results indicate the current label withdrawal period of 12 d for flunixin meglumine after a single IM administration at 2.2 mg/kg should be sufficient for finishing-age swine, and untreated pigs can be exposed to flunixin by treated pigs due to environmental contamination. By integrating the experimental data with PBPK modeling, another significant finding is that plasma and urine concentrations may be useful markers to predict tissue residue statuses of flunixin after IM administration in swine by using a PBPK model.

### Correlation of urine flunixin concentrations with tissue residue depletion profiles

The PBPK model was employed to predict flunixin concentrations in the urine to assess the potential for using the model to predict residue depletion profiles in edible tissues based on less invasive and easily accessible biological matrices. The model-predicted data were compared to the observed data in spot urine samples collected at slaughter time points (Fig. 1d). Notably, the model-predicted urine flunixin concentrations at 24 h and 96 h were both in the range of observed values, although concentrations at 24 h varied greatly. Specifically, the model-predicted value at 24 h was similar to the measured values in two of the three animals, but was lower than that of the third animal by nearly threefold. This is not surprised as spot urine concentrations of a drug depend on multiple factors, including urinary output, voiding interval time, previous voiding time, and postvoid residual urine volume [29]. As a result, the underestimation of the third animal could be the result of smaller urinary output, longer voiding interval time, and/or larger postvoid residual urine volume than two other animals. Nonetheless, adequate predictions of spot urine flunixin concentrations suggest that the PBPK model can potentially be used to perfrom reverse dosimetry analysis, i.e., to predict the tissue residue depletion profiles based on the experimentally-measured spot urine concentration data. Future studies should consider using metabolism cages to collect cumulative urine samples to further refine this PBPK model.

### Assessment of flunixin exposure in untreated pigs due to environmental contamination

At Days 1 and 4 after treatment with sterile water vehicle, control pigs had no detectable concentrations of flunixin or 5-hydroxyflunixin in the plasma (Tables 1 and 2) or tissue samples (Tables 3 and 4). However, flunixin was found in their urine samples by liquid chromatography with tandem mass spectrometry (LC-MS/MS) testing at Days 1 and 4 following vehicle treatment (Table 5). The detection of flunixin at higher concentrations in urine and not in plasma was not unexpected based on earlier studies in horses [26, 30]. Low oral bioavailability of FM in swine [19] could partially explain why environmental exposure to contaminated urine would be insufficient to result in detectable tissue residues, which was confirmed in current study. On all necropsy days after Day 4, none of the vehicle-treated control pigs had quantifiable flunixin or 5-hydroxyflunixin residues in plasma, urine, or tissue samples.

### Examine the effectiveness of determining plasma, urine and oral fluid flunixin concentrations as potential markers of tissue residue status in pigs

To assess the effectiveness of oral fluids as a potential biomarker for predicting tissue residues, the PBPK model was extended to predict flunixin concentrations in oral fluid. The results showed that PBPK model-predicted levels were in the range of the observed concentrations across all slaughter time points, with good correspondence at 24 h (Fig. 1e). However, the measured data at 36 h and 48 h was highly variable, and the model simulations were at the lower end of the range (Fig. 1e). These data indicate that oral fluids at 24 h could be a potentially useful biological matrix for estimating plasma and tissue residue levels of flunixin by utilizing this PBPK model. However, further studies with individual animal data on oral fluid samples at 36 and 48 h are needed to optimize the model.

Parent flunixin showed in urine of all pigs on Day 4 and in the pen-level oral fluids through Day 12 (Table 5), but flunixin did not show in pen-level oral fluids samples on Day 8 after treatment. Additionally, 5-hydroxyflunixin was found in all pen-level oral fluids samples through Day 16, except on Day 8. The reason of the absence of parent flunixin and the metabolite 5-hydroxyflunixin concentrations on Day 8 was unknown, but could relate to sample collection methods or the way in which FM-treated and vehicle-treated control pigs interact with the rope at the pen level. Although analysis of oral fluids could be useful in revealing drug exposure, particularly regarding the assessment of drug metabolites, additional studies in individual and group-housed pigs are needed to improve oral fluids collection method for drug analysis.

### Adequacy of the current label withdrawal period of 12 days of flunixin meglumine in pigs

Beyond 24 h after drug treatment, flunixin levels in all studied tissue samples were less than the limit of quantitation of the analytical method (LOQ = 5 ng/g), except in the injection site of one pig at 4 d and one pig at 12 d post treatment and in the muscle of one pig at 16 d post treatment (Table 3). It is important to note that flunixin levels in all tissues after Day 12 (the labeled tissue withdrawal period for FM in swine) fell below established tolerance in swine (30 and 25 ng/g in liver and muscle, respectively) [28] and therefore indicate no significant food safety concerns. Also, 5-hydroxyflunixin was not seen in any of the studied tissues after 24 h (Table 4).

### Limitations of this study

The present study has several limitations. The number of animals was only three per sampling time. This is insufficient to characterize animal population variability. For a tissue residue depletion study designed to determine withdrawal period, at least four (evenly mixed as per sex) animals each slaught time (ideally 5 each time point) are recommended, according to the guidelines from U.S. Food and Drug Administration [31, 32]. Also, concentrations of flunixin and 5-hydroxyflunixin in the edible tissues were found only at one sampling time (i.e., Day 1), insufficient to characterize the tissue residue depletion profiles. Additional studies using a larger number of animals and including sampling times from Day 1 to Day 4 are needed to better characterize the depletion profile of flunixin in finishing-age swine. In addition, the PBPK model used in this study was based on a previously published PBPK model developed in cattle using a legacy software program (acslX) that was discontinued. As a quantitative tool, this model is sufficient to demonstrate the correlation of plasma, urine and tissue concentrations of flunixin. However, for future application, readers are advised to use the recently published more comprehensive PBPK model for flunixin in cattle and swine developed in Berkeley Madonna and was translated to a user-friendly, web-based interactive PBPK (iPBPK) interface using R Shiny [33].

## Conclusions

The present study reports original data on the depletion kinetic profiles of flunixin and its metabolite 5-hydroxyflunixin in the plasma, urine, liver, kidney, muscle, injection site, and oral fluid samples in finishing-age pigs after IM administration of FM at 2.2 mg/kg. A PBPK model in pigs was adapted based on the collected data and a previously published model in cattle [27]. The model adequately correlates plasma, oral fluid and urine concentrations of flunixin with the residue depletion profiles in the liver, kidney, and muscle of finishing-age swine, especially within 24 h after dosing. Plasma, urine, and oral fluid flunixin concentrations can be useful biomarkers for predicting tissue residues in pigs at earlier time points (≤24 h) by using a PBPK model, results of this study suggest. Oral fluids and urine together with a PBPK model can be less invasive and more easily administered antemortem biological monitoring tools for assessing tissue exposure to drugs, especially during the first 24 h after drug exposure. However, this would limit the utility of this system in swine production systems where samples are seldom collected before the end of the label tissue withdrawal period. This system may have more applicability to drugs with longer plasma elimination half-lives that could be found beyond 24 h after administration.

## Methods

All animal procedures described in this manuscript were approved by the Iowa State University Animal Care and Use Committee (IACUC # 3-14-7768-S) before starting this experiment.

### Animals

Twenty crossbred barrows (mean body weight: 128.2 kg [282.6 lbs]) were purchased from a commercial swine finishing system (Audubon Manning Veterinary Clinic Management Services, Audubon, IA). None of the twenty pigs had records of prior treatment with flunixin meglumine or other nonsteroidal anti-inflammatory drugs (NSAID). Each pig was confirmed healthy by physical examination. Inclusion criteria included good clinical health with no prior administration of flunixin meglumine. Administration of flunixin meglumine prior to this study excluded any pig from enrollment into this study. Each enrolled pig received a numerical ear tag (Allflex Global Ear Tags, Allflex USA, Inc., DFW Airport, TX, USA) in the right ear and was weighed. A 1-in. diameter, circular tattoo was placed in each pig on the left side in the post-auricular area, around 2 in. ventral to the dorsal midline and 2 in. caudal to the ear. This tattoo was applied on the skin above the trapezius muscle using a commercial tattoo applicator (Stone Mfg., Kansas City, MO, USA) with a slap tattoo. The pigs also had a blood draw to obtain serum to check for pre-treatment levels of flunixin and 5-hydroxyflunixin using the LC-MS/MS method described below.

Pigs were housed in Building 29 of Veterinary Medical Research Institute Facilities at the Iowa State University. The pigs were weighed on arrival and blocked by weight prior to random allocation into treatment groups using a random number generator (Microsoft Excel, Redmond, WA) according to anticipated necropsy date. Five housing groups of four pigs each group were determined and placed in separate rooms based on the planned necropsy date and treatment group (Table S1). In this pharmacokinetic study, there were no statistical hypotheses being tested. Therefore, classical statistical methods (e.g., power analysis) used to calculate sample size were not relevant. However, in this study the sample size of *n* = 4 pigs per group each necropsy date was based on principles outlined in the book of Comparative Pharmacokinetics - Principles, Techniques, and Applications: “*A broad examination of the comparative pharmacokinetic literature suggests that the typical size of an intravenous dose pharmacokinetic trial for a drug with normal variability in the population is approximately four to six animals*” [34].

Animal care and housing conditions were in compliance with the guidelines outlined in the Guide for the Care and Use of Agricultural Animals in Agricultural Use and Research and Teaching 3rd Edition [35]. Pigs were hand-fed once per day with an organic corn/soybean meal diet that met the National Research Council (NRC) nutrient requirements for finishing pigs [36], and were confirmed free of any medications. Pigs had open access to water through a nipple waterer throughout the study.

### Animal phase study design and sample collection

The body weight of each pig was measured the day before treatment to determine accurate dosage. One pig of each housing group was designated the negative control using a random number generator (Microsoft Excel, Redmond, WA), while the other three pigs in the housing group were treated with FM. On the first day of the experiment, each animal was restrained with a hog snare and had a blood sample drawn just before treatment, then each treated pig was given 2.2 mg/kg flunixin meglumine (FM, Banamine-S, Merck Animal Health, Lot # 3037102, Expiration Date: 2/2015) and control pigs were given an equivalent volume of sterile water (VetOne Sterile Water, Nova-Tech, Inc., Grand Island, NE, USA, Lot # B131107–2, Expiration Date: 11/2015). This dose was given IM with a 16-gauge, 1-in. needle inside circular tattoo placed on arrival in the post-auricular area. The information on pig weights and specific treatment given is provided in Table S1 (Supplementary Material).

Blood samples of the pigs (8 mL each sample) were collected at 0, 1, 3, 6, 12, 24, 36 and 48 h after treatment with FM or sterile water via the left or right jugular vein using a 25.4 mm 16-gauge hypodermic needle (Air-Tite Products, Virginia Beach, VA, USA) and 12 mL Luer lock syringe (TycoHealth Care, Mansfield, MA, USA). In addition, just before necropsy for each group, the pigs had a final blood draw. During blood sample collection, animals were restrained manually using a pig snare. To collect oral fluid samples, a ½-inch diameter cotton rope was suspended from a hook hanging to the pig’s shoulder height for around 20–30 min of sampling time to allow each pig to chew on the rope, and then the rope was removed. Oral fluids were extracted by placing the wet portion of the rope into a clean plastic bag and squeezing the rope so the fluid accumulates in the bag, with a minimum of 5 mL being considered as acceptable. Fluids were poured into a cryovial and stored at − 80 °C before analysis.

Necropsy and tissue sample collection took place at 1, 4, 8, 12, and 16 d after treatment with FM or sterile water. Just before euthanasia and necropsy, a 8-mL blood sample were collected at times according to the initial treatment time on Day 1. Pigs were euthanized by penetrating captive bolt followed by exsanguination according to American Veterinary Medical Association guidelines [37]. Urine and tissue samples, including the injection site, liver, kidney, semitendinosus/semimembranosus muscle were collected and stored at − 80 °C before analysis.

### Sample processing and analysis

The Iowa State University Veterinary Diagnostic Lab and the Iowa State University-Pharmacology Analytical Support Team (ISU-PhAST) analyzed concentrations of flunixin and its metabolite 5-hydroxyflunixin in plasma, urine, oral fluid, and tissue samples (i.e., liver, kidney, muscle, and injection site). All individuals performing laboratory analysis were blinded to treatment groups. Laboratory samples were labeled in a coded manner, making the treatment status of sample unknown to the laboratory. Blood for analysis was collected in a 10-mL heparinized blood collection tube (BD Vacutainer, Franklin Lakes, NJ, USA) and then centrifuged for 10 min at 1500 g. The plasma was collected and immediately frozen and stored at − 80 °C. Plasma samples were analyzed for flunixin and 5-hydroxyflunixin within 60 d after sample collection and within 10 consecutive days once analysis was started. Urine was collected at necropsy with tissue collection. Three mL of urine was aspirated from the urinary bladder using a 3-mL Luer Lock syringe with an attached 22-gauge × 3/4-in. needle. This sample was placed in a non-additive red top tube and stored at − 80 °C before analysis.

Standards were prepared at a final working concentration ranging from 5 to 5000 ng/mL flunixin and 5-hydroxyflunixin. Internal standard (i.e., flunixin D-3) was added to samples and standards. Oral fluid samples were buffered to a pH of 2.9–3.0; and then flunixin and 5-hydroxyflunixin were extracted using methyl tert-butyl ether. Urine samples were prepared by diluting 0.5 mL urine with water, and then a base catalyzed hydrolysis was performed at the room temperature for 15 min. The diluted urine samples were buffered to a pH of 2.9–3.0 and extracted using 10:1 dichloromethane:petroleum ether. For both oral fluid and urine samples, the organic layer was transferred, dried down and reconstituted in 12.5% acetonitrile in water. Plasma samples were diluted with acetonitrile, centrifuged, and then the supernatant was transferred and dried down. Drugs from tissue samples (liver, kidney, muscle, and injection site) were analyzed using an FSIS method (CLG-MRM 1.02) [38] with a few modifications. In brief, flunixin and 5-hydroxyflunixin were extracted in 80% (v/v) acetonitrile in water and then further purified using Bakerbond (C18). Both plasma and tissue samples were reconstituted in 25% acetonitrile in water. The recovery of the extraction method for flunixin and 5-hydroxyflunixin from tissues and biological fluids were not measured. However, quality control samples were utilized to ensure that extractions were consistent, precise and reproducible. All samples were transferred to an autosampler vial (with glass insert) and centrifuged before analysis with LC-MS/MS.

A TSQ Quantum Discovery Max triple quadrupole mass spectrometer was used to analyze concentrations of flunixin and 5-hydroxyflunixin in oral fluids and urine. The three ions used in positive ion mode were (m/z) 297 → 109/264/279 for flunixin and (m/z) 313 → 109/280/295 for 5-hydroxyflunixin. Concentrations of flunixin and 5-hydroxyflunixin in plasma samples were measured utilizing an ABSciex QTRAP 4500 mass spectrometer. The three ions measured in negative ion mode were (m/z) 295 → 251, 210, and 197 for flunixin and (m/z) 311 → 267, 227, and 247 for 5-hydroxyflunixin. All curves had a coefficient of determination > 0.99. Limit of quantification (LOQ) for this assay was 5 ng/g or ng/mL for both flunixin and 5-hydroxyflunixin in plasma, oral fluid, tissue, and urine samples. Limit of detection was 3 ng/mL in plasma and 1 ng/mL in oral fluids for both chemicals, and was 2 ng/mL for flunixin and 3 ng/mL for 5-hydroxyflunixin in urine. Plasma samples were run for accuracy and interday and intraday precision. Due to lower sample volume, urine/oral fluids/tissues were each analyzed in a single run so accuracy and interday precision was monitored. Data were accepted only if quality control samples for each run were within 20% of expected nominal levels.

### Pharmacokinetic analysis

A PBPK model for flunixin in finishing-age pigs was developed via extrapolation from a published PBPK model for flunixin in cattle [27] and was re-calibrated with the newly collected experimental data from the present study. The model consisted of eight compartments (blood, liver, kidneys, muscle, fat, lungs, richly and slowly perfused tissues) for flunixin and a one-compartment sub-model for pooled metabolites (Figure S1, Supplementary Material). IM injection was simulated as a first-order absorption process with a two-compartment model. The distribution in all compartments was assumed to be blood flow-limited (i.e., perfusion-limited model). The elimination pathways included hepatic metabolism, biliary excretion via liver, and urinary excretion by kidney. The model was updated to include the salivary excretion pathway to assess the potential correlation between plasma, oral fluid and urine levels of flunixin and the residue depletion profiles in edible tissues. Oral fluid concentration of flunixin was defined as plasma level multiplied by saliva/plasma partition coefficient [39], which was calculated using the mean saliva/plasma area under the concentration ratio method. All physiological parameters were updated to be pig-specific [40, 41]. All chemical-specific parameters remained the same except the absorption parameters, which were estimated by fitting with the newly generated pharmacokinetic data for flunixin in finishing-age pigs from the present study using the Nelder-Mead optimization algorithm in acslX (AEgis Technologies, Inc., Huntsville, AL, USA). All pig-model specific parameters are shown in Table S2 (Supplementary Material). Other parameters and detailed description about model calibration and parameterization processes refer to the original publication [27] and our recent review article on the principles and methodology of PBPK modeling in veterinary medicine [42].

## Supplementary information

**Additional file 1: Table S1.** on the study animal weights, necropsy group allocations and treatment information; **Table S2.** on the parameters used in the PBPK model for flunixin in finishing-age pigs; and **Figure S1.** on the PBPK model schematic.

## Data Availability

All raw experimentally measured concentration data of flunixin and its metabolite 5-hydroxyflunixin in plasma, liver, kidney, muscle, injection site, urine, and oral fluid are presented in Tables 1-5. The PBPK model code in the legacy software format of acslX is available upon request from the corresponding author. Readers are referred to obtain a more recent version of the PBPK model in Berkeley Madonna that has been translated to a web-based interactive PBPK (iPBPK) interface as described in Li et al. [33].

## Abbreviations

FM: Flunixin meglumine
IM: Intramuscular
iPBPK: Interactive physiologically based pharmacokinetic
IV: Intravenous
LC-MS/MS: Liquid chromatography with tandem mass spectrometry
LOQ: Limit of quantification
NRC: National research council
NSAID: Nonsteroidal anti-inflammatory drug
PBPK: Physiologically based pharmacokinetic
