# Supplementary material for: A study to assess the correlation between plasma, oral fluid and urine concentrations of flunixin meglumine with the tissue residue depletion profile in finishing-age swine
Source: BMC Vet Res. 2020 Jun 22;16:211. doi: 10.1186/s12917-020-02429-w (PMC7310148; doi:10.1186/s12917-020-02429-w)
Supplement: Supplementary file 1 — Additional file 1: Table S1. on the study animal weights, necropsy group allocations and treatment information; Table S2. on the parameters used in the PBPK model for flunixin in finishing-age pigs; and Figure S1. on the PBPK model schematic. [file 12917_2020_2429_MOESM1_ESM.docx]

**Supplementary Material**

A study to assess the correlation between plasma, oral fluid and urine concentrations of flunixin meglumine with the tissue residue depletion profile in finishing-age swine

Jessica L. Bates^1^, Locke A. Karriker^1^, Suzanne M. Rajewski^2^, Zhoumeng Lin^3,*^, Ronette Gehring^3^, Mengjie Li^3^, Jim E. Riviere^3^, Johann F. Coetzee^2,3^

^1^ Swine Medicine Education Center, Veterinary Diagnostic and Production Animal Medicine, College of Veterinary Medicine, Iowa State University, Ames, Iowa, 50011, USA

^2^ Analytical Chemistry Services, Veterinary Diagnostic and Production Animal Medicine, College of Veterinary Medicine, Iowa State University, Ames, Iowa, 50011, USA

^3^ Institute of Computational Comparative Medicine (ICCM), Department of Anatomy and Physiology, College of Veterinary Medicine, Kansas State University, Manhattan, KS 66506, USA

* Correspondence should be addressed to Zhoumeng Lin: Tel, +001-785-532-4087; Fax, +001-785-532-4953; E-mail, zhoumeng@ksu.edu. Institute of Computational Comparative Medicine (ICCM), Department of Anatomy and Physiology, College of Veterinary Medicine, Kansas State University, 1800 Denison Avenue, P200 Mosier Hall, Manhattan, KS 66506, USA.

**Present address**: Ronette Gehring, Institute for Risk Assessment Sciences, Division of Toxicology and Pharmacology, Utrecht University, Utrecht, The Netherlands. Mengjie Li, Department of Pharmaceutical Sciences, School of Pharmacy and Pharmaceutical Sciences, University at Buffalo, Buffalo, NY 14260, USA.

**Table S1**. Study animal weights, necropsy group allocations and treatment information.

| **Group** | **Pig ID** | **Weight (kg)** | **Injection Volume (mL)** |
| --- | --- | --- | --- |
| **G1**  **(Day 1)** | **260** | 125.00 | 5.5 |
|  | **261** | 133.64 | 5.9 |
|  | **270 (C)** | 123.64 | 5.4 |
|  | **273** | 136.36 | 6.0 |
| **G2**  **(Day 4)** | **262** | 138.64 | 6.1 |
|  | **264** | 128.18 | 5.6 |
|  | **269** | 133.64 | 5.9 |
|  | **274 (C)** | 136.36 | 6.0 |
| **G3**  **(Day 8)** | **258** | 134.09 | 5.9 |
|  | **266 (C)** | 130.45 | 5.7 |
|  | **268** | 134.55 | 5.9 |
|  | **272** | 139.09 | 6.1 |
| **G4**  **(Day 12)** | **263** | 143.18 | 6.3 |
|  | **267** | 132.73 | 5.8 |
|  | **271** | 128.18 | 5.6 |
|  | **275 (C)** | 131.82 | 5.8 |
| **G5**  **(Day 16)** | **257** | 130.00 | 5.7 |
|  | **259** | 134.09 | 5.9 |
|  | **265 (C)** | 126.82 | 5.6 |
|  | **276** | 138.18 | 6.1 |

**Note**: Treated pigs received a single dose of 2.2 mg/kg flunixin meglumine (Banamine S, Merck Animal Health) intramuscularly (IM) on Day 0. Untreated, control pigs used to assess the potential for environmental contamination were designated (C) and received an equivalent volume of sterile water (VetOne Sterile Water, Nova-Tech, Inc.).

| **Table S2**. Parameters used in the PBPK model for flunixin in finishing-age pigs. | | | | |
| --- | --- | --- | --- | --- |
| Parameter | Description | Value | Unit | Source |
| *Weight and volume^a^* | |  |  |  |
| BW | Body weight | 132.93 | Kg | Measured^b^ |
| FVBlood | Fraction of BW as blood | 0.06 | Unitless | Buur et al. (2005), Upton (2008) |
| FVven | Venous fraction of blood | 0.74 | Unitless | Buur et al. (2005), Upton (2008) |
| FVF | Fraction of BW as fat | 0.32 | Unitless | Buur et al. (2005), Upton (2008) |
| FVK | Fraction of BW as kidneys | 0.004 | Unitless | Buur et al. (2005), Upton (2008) |
| FVL | Fraction of BW as liver | 0.0247 | Unitless | Buur et al. (2005), Upton (2008) |
| FVLu | Fraction of BW as lungs | 0.01 | Unitless | Buur et al. (2005), Upton (2008) |
| FVM | Fraction of BW as muscle | 0.4 | Unitless | Buur et al. (2005), Upton (2008) |
| FVP | Fraction of BW as poorly perfused tissues excluding fat and muscle | 0.1109 | Unitless | Buur et al. (2005), Upton (2008) |
| FVR | Fraction of BW as richly perfused tissues excluding blood, liver, kidneys and lungs | 0.0704 | Unitless | Buur et al. (2005), Upton (2008) |
| Hematocrit | Erythrocyte volume fraction | 0.33 | Unitless | Buur et al. (2005), Upton (2008) |
| *Blood flow rate^a^* | |  |  |  |
| QCC | Cardiac output | 5 | L/h/kg | Upton (2008) |
| FQR | Fraction QCC to richly perfused tissues excluding liver and kidneys | 0.0955 | Unitless | Buur et al. (2005), Upton (2008) |
| FQP | Fraction QCC to slowly perfused tissues excluding fat and muscle | 0.1335 | Unitless | Buur et al. (2005), Upton (2008) |
| FQK | Fraction QCC to kidneys | 0.12 | Unitless | Buur et al. (2005), Upton (2008) |
| FQL | Fraction QCC to liver | 0.2725 | Unitless | Buur et al. (2005), Upton (2008) |
| FQF | Fraction QCC to fat | 0.1275 | Unitless | Buur et al. (2005), Upton (2008) |
| FQM | Fraction QCC to muscle | 0.251 | Unitless | Buur et al. (2005), Upton (2008) |
| Qurine | Urinary output | 0.097 | L/h | Gans and Mercer (1977) |
| *Partition coefficient* | |  |  |  |
| PK | Unbound kidney tissue to plasma | 300 | Unitless | Estimated^c^ |
| Psaliva/plasma | Saliva to plasma | 3.5 | Unitless | Calculated^b^ |
| *Absorption parameter* | |  |  |  |
| Kim | Intramuscular absorption rate constant | 2 | h^-1^ | Optimized^d^ |
| Ksite12 | First order transport rate constant from absorption site 1 to site 2 | 2 | h^-1^ | Optimized^d^ |
| Ksite21 | First order transport rate constant from absorption site 2 to site 1 | 0.00008 | h^-1^ | Optimized^d^ |
| ^a^ All physiological parameter values represent the average of values from Buur et al. (2005) [1] and Upton (2008) [2], except QCC that is from Upton (2008) [2] and Qurine is from Gans and Mercer (1977) [3]. All other model parameters are the same as those in the flunixin PBPK model in cattle [4].  ^b^ These parameters were measured or calculated based on data from the present study. | | | | |
| ^c^ This parameter was estimated by manually fitting to the measured kidney data from the current study. | | | | |
| ^d^ These parameters were optimized by fitting to the measured plasma data from this study using the Nelder-Mead method in Acslx^TM^. Ksite21 was further optimized manually to fit the plasma data. | | | | |


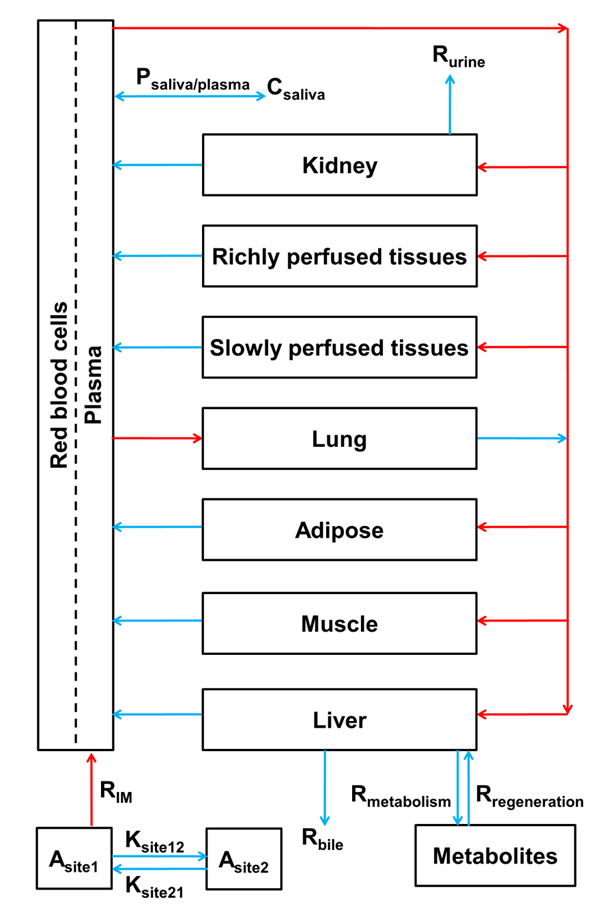


**Figure S1**. A schematic diagram for a physiologically based pharmacokinetic (PBPK) model for flunixin in finishing pigs. A_site1_ and A_site2_ [µg] represent the amounts of flunixin in the absorption site 1 and site 2, respectively. K_site12_ and K_site21_ [h^-1^] are first order transport rate constants from absorption site 1 to site 2 and from site 2 to site 1, respectively. R_bile_ and R_urine_ [µg/h] stand for biliary and urinary excretion rates, respectively. C_saliva_ [ng/ml], flunixin concentration in the oral fluids; P_saliva/plasma_ [unitless], saliva/plasma flunixin partitioning coefficient; R_IM_ [µg/h], intramuscular absorption rate; R_metabolism_ [µg/h], hepatic metabolic rate of flunixin; R_regeneration_ [µg/h], regeneration rate of flunixin from metabolites due to both enterohepatic recirculation and hydrolysis of conjugates.

**References**:

1. Buur JL, Baynes RE, Craigmill AL, Riviere JE: Development of a physiologic-based pharmacokinetic model for estimating sulfamethazine concentrations in swine and application to prediction of violative residues in edible tissues. *Am J Vet Res.* 2005, 66(10):1686-1693.

2. Upton RN: Organ weights and blood flows of sheep and pig for physiological pharmacokinetic modelling. *J Pharmacol Toxicol Methods.* 2008, 58(3):198-205.

3. Gans JH, Mercer PF: Chapter 37: The Kidneys. In: *Dukes Physiology of Domestic Animals.* 9th edn. Edited by Swenson MJ. Ithaca, NY: Cornell University Press; 1977: 463-492.

4. Leavens TL, Tell LA, Kissell LW, Smith GW, Smith DJ, Wagner SA, Shelver WL, Wu H, Baynes RE, Riviere JE: Development of a physiologically based pharmacokinetic model for flunixin in cattle (Bos taurus). *Food Addit Contam Part A Chem Anal Control Expo Risk Assess.* 2014, 31(9):1506-1521.
